# Open Access Publishing of Textbooks and Guidelines for Otolaryngologists in Developing Countries

**DOI:** 10.1177/2473974X19861567

**Published:** 2019-07-10

**Authors:** Johannes J. Fagan

**Affiliations:** 1 Division of Otolaryngology, University of Cape Town, Cape Town, South Africa

**Keywords:** open access, publishing, medical, developing world, otolaryngology, ENT, guidelines, surgery atlas, audiology

## Abstract

Accessing educational and scientific material is key to improving otolaryngology care in developing countries. Yet current financial models of publishers restrict access to academic information. This article describes the author’s experience with self-publishing 2 open access textbooks, *Open Access Atlas of Otolaryngology, Head and Neck Operative Surgery* and *Open Access Guide to Audiology and Hearing Aids for Otolaryngologists*, as well as the *African Head and Neck Society (AfHNS) Clinical Practice Guidelines for Head and Neck Cancer in Developing Countries and Limited Resource Settings*. The author outlines the simplicity, advantages, and popularity of this form of publication and why societies and individuals should embrace open access publishing to benefit especially those studying and practicing in developing countries. He discusses some of the challenges related to open access publishing and calls for medical societies to become involved in evaluating the quality of open access texts and videos for their members.

Accessing educational and scientific material is key to improving otolaryngology (ENT) care in developing countries. Yet many trainees, practitioners, and researchers cannot afford textbooks and pay-to-view journals. The author self-publishes 2 open access textbooks, *Open Access Atlas of Otolaryngology, Head and Neck Operative Surgery* (*OAA*) and *Open Access Guide to Audiology and Hearing Aids for Otolaryngologists*. More than 100 authors from >20 countries have contributed, and volunteers have translated chapters into Portuguese, Spanish, and French. Recently, the African Head and Neck Society (www.afhns.org) launched another open access resource, the *African Head and Neck Society (AfHNS) Clinical Practice Guidelines for Head and Neck Cancer in Developing Countries and Limited Resource Settings*. This article describes the author’s experience with these open access resources.

## The *OAA*

**Figure 1.**
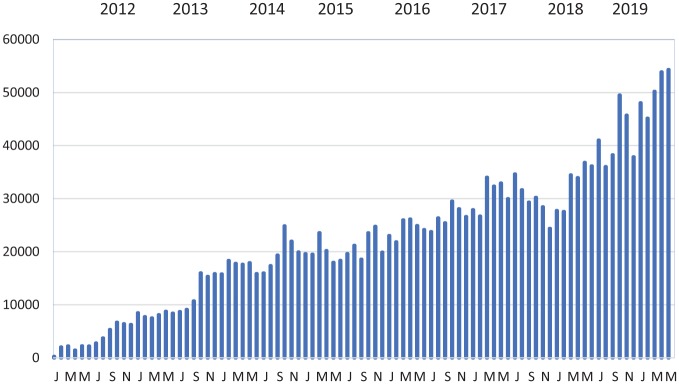
*Open Access Atlas* chapters downloaded per month.

The *OAA* (http://www.entdev.uct.ac.za/guides/open-access-atlas-of-otolaryngology-head-neck-operative-surgery) provides detailed descriptions of surgical procedures. The authors are mostly international leaders who volunteered to contribute. It includes surgical procedures no longer performed in developed countries such as hammer and gouge mastoidectomy, which would not be included in modern textbooks. Being in electronic format, chapters are very detailed with numerous photographs and videoclips. Chapters are still being added, and existing chapters are edited from time to time. This illustrates an important advantage of electronic textbooks: they do not have to be completed before being published, as chapters can be added over time. **Figure 1** illustrates the growing popularity of the *OAA*: as additional chapters are added, it becomes better known and translations are added. The *OAA* received the Open Education Consortium 2017 Award for Open Education Excellence, a tribute to its contributors (http://www.oeconsortium.org/projects/open-education-awards-for-excellence/2017-oe-award-winners-oer-categories/).

## Audiology and Hearing Aid Fitting for Otolaryngologists

**Figure 2.**
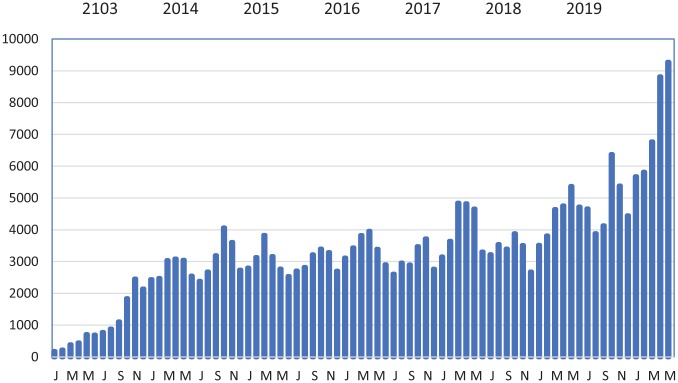
*Open Access Guide to Audiology* chapters downloaded per month.

Only 4 countries in sub-Saharan Africa train audiologists. Hence, audiology and hearing aid fittings are generally done by ENTs, as occurs in some central European countries.^1,2^ This textbook, *Open Access Guide to Audiology and Hearing Aids for Otolaryngologists* (http://www.entdev.uct.ac.za/guides/open-access-guide-to-audiology-and-hearing-aids-for-otolaryngologists), aims to improve audiologic care by ENTs. **Figure 2** illustrates its growing popularity.

## Publishing Process

Chapters are submitted in Microsoft Word format, and the author (J.J.F.) edits, formats, and uploads a PDF version to the University of Cape Town webserver. The textbooks are registered with the Creative Commons, a nonprofit corporation that issues public copyright licenses (https://creativecommons.org). Readers may use material provided it is referenced. Hyperlinks to individual chapters are maintained on the IFOS Developing World ENT website (www.entdev.uct.ac.za.guides/).

## Popularity

Compared with big ENT textbooks that sell about 4000 copies, chapters have been downloaded >2.3 million times, currently 2100 downloads per day (ie, every 41 seconds). Although intended for developing countries, 8 of the 10 top users are developed countries (**Table 1**).

**Table 1.** Two Developing Countries (Shaded) Are among the Top 10 Users of the Textbooks.

Rankings	Countries
1	United States
2	India
3	United Kingdom
4	South Africa
5	Spain
6	France
7	Netherlands
8	Saudi Arabia
9	Italy
10	Sweden

## Popular Chapters

Editors have little idea what readers wish to read when planning a textbook. However, online textbooks, of which chapters are individually downloaded, provide such data (**Table 2**). Surprisingly, pectoralis major flap is the most popular chapter.

**Table 2.** Ten Most Popular Chapters in *Open Access Atlas*.

Rankings	Chapters
1	Pectoralis major flap
2	Submandibular gland excision
3	Parotidectomy
4	Ranula and sublingual salivary gland excision
5	Modified and radical neck dissection technique
6	Total laryngectomy
7	Thyroidectomy
8	Surgical drainage of deep neck abscesses
9	Mastoidectomy and epitympanectomy
10	Myringoplasty and tympanoplasty

## Open Access Textbooks as Revenue Generator

Even though it is tempting to monetize open access textbooks to raise funds for worthy causes, this was not done, as the authors contributed with the understanding that it is a philanthropic project.

## AfHNS Clinical Practice Guidelines for Head and Neck Cancer in Developing Countries and Limited Resource Settings

International guidelines have limited value in limited resource settings because of lack of cytology, imaging, (chemo)radiation, complex surgery, and even thyroid and calcium replacement after thyroidectomy. Clinicians in the “Global South” should therefore develop resource-appropriate guidelines for developing countries, rather than be guided by institutions in the “Global North,” as they know best what the challenges, limitations, and possibilities are to deliver care. The AfHNS (www.afhns.org) launched this novel open access resource in 2019. African surgeons and oncologists formulated the guidelines (https://developingworldheadandneckcancerguidelines.com/) in consultation with American and European colleagues to ensure resource-appropriate best care for patients in developing countries. Thyroid, parotid, and submandibular gland guidelines have been released and are precisely tailored to availability of diagnostic investigations and therapeutic interventions. Guidelines for other head and neck sites will be released in the future. This ability to self-publish web-based guidelines permits countries in the “Global South” to assume leadership and take responsibility for their own regions without having to work through established institutions or publishers. That there were 1630 visitors and 7860 page views of the guidelines in the first 3 months demonstrates the power of web-based publishing.

## Why Adopt an Open Access Policy?

Apart from the benefits previously described, reasons why societies and individual authors should adopt an open access policy for educational material include the following:

- Our open access texts have not cost a cent to produce
- Authors retain copyright
- Publishers cannot discontinue publications
- Chapters can be edited/updated, even on a mobile phone
- Reaches remote areas including conflict zones where textbooks are inaccessible
- Niche topics can be included
- Can be read on a mobile phone
- Electronic texts will become translatable into many languages

## Conclusions

The author’s experience demonstrates how open access publishing is a great leveler across developed and developing worlds by making educational material freely available and empowering one to bypass established publishers and institutions.

However, open access publishing presents challenges such as the pay-to-publish model, a barrier for authors from developing countries, and concerns about the quality of predatory journals. Just as governments are regulating social media, medical societies should identify reputable YouTube videos, textbooks, and journals for their membership.

## Author Contributions

**Johannes J. Fagan**, conceived, designed, and authored the article.

## Disclosures

**Competing interests:** None.

**Sponsorships:** None.

**Funding source:** None.
